# DNA Fingerprinting of *Mycobacterium tuberculosis*: Lessons Learned and Implications for the Future

**DOI:** 10.3201/eid0811.020402

**Published:** 2002-11

**Authors:** Scott J. N. McNabb, Christopher R. Braden, Thomas R. Navin

**Affiliations:** * Centers for Disease Control and Prevention, Atlanta, Georgia, USA

**Keywords:** *Mycobacterium tuberculosis*, DNA fingerprinting, RFLP typing, restriction fragment length polymorphism, tuberculosis

## Abstract

DNA fingerprinting of *Mycobacterium tuberculosis*—a relatively new laboratory technique—offers promise as a powerful aid in the prevention and control of tuberculosis (TB). Established in 1996 by the Centers for Disease Control and Prevention (CDC), the National Tuberculosis Genotyping Surveillance Network was a 5-year prospective, population-based study of DNA fingerprinting conducted from 1996 to 2000. The data from this study suggest multiple molecular epidemiologic and program management uses for DNA fingerprinting in TB public health practice. From these data, we also gain a clearer understanding of the overall diversity of *M. tuberculosis* strains as well as the presence of endemic strains in the United States. We summarize the key findings and the impact that DNA fingerprinting may have on future approaches to TB control. Although challenges and limitations to the use of DNA fingerprinting exist, the widespread implementation of the technique into routine TB prevention and control practices appears scientifically justified.

The capacity to differentiate *Mycobacterium tuberculosis* strain patterns by DNA fingerprinting has shown promise in tuberculosis (TB) control since this tool was first applied to outbreak investigations (1–3) and population-based studies (4,5) in the early 1990s. Evaluating this tool and determining its limitations are important activities in view of the most recent efforts to eliminate TB in the United States after its resurgence in the early 1990s (6). In 1996, the Centers for Disease Control and Prevention (CDC) initiated a 5-year, prospective, population-based study, the National Tuberculosis Genotyping Surveillance Network. Most findings represented in this issue of Emerging Infectious Diseases come from this study. In this synopsis, we address two important implications for DNA fingerprinting of *M. tuberculosis*: its varied utility as a tool in TB prevention and control and its value in the measurement of the overall diversity of *M. tuberculosis* strain patterns in the United States, including differences by region and population and the prevalence of endemic strains.

## Identification of Laboratory Cross-Contamination or Mislabeling

DNA fingerprinting of *M. tuberculosis* has been shown to identify and confirm laboratory cross-contamination or mislabeling. Previous retrospective studies describing *M. tuberculosis* laboratory cross-contamination or mislabeling found rates between 0.9% and 3.5% (7–14). In this issue, Northrup et al. (15) report a rate of 1.5%, which is within the range of published rates. Therefore, of 13,035 culture-positive TB case-patients reported in the United States in 2000, TB may have been misdiagnosed in as many as 117 (0.9%) to 456 (3.5%) persons. Using the previously reported finding that two thirds of case-patients with false-positive cultures are treated (11), we estimate that 78–304 persons may have been misdiagnosed and treated unnecessarily in the United States in 2000.

To measure the direct and indirect financial costs associated with these laboratory cross-contamination or mislabeling events, Northrup et al. (15) discuss data about three persons who were falsely diagnosed as having TB. In 1999 U.S. dollars, the estimated average cost to the health-care system for each person was $32,231 and the estimated average direct cost for each person was $10,744; thus, the estimated average cost for misdiagnosis of TB was $42,975 per person. This study did not include indirect and intangible costs, which can be substantial and are largely paid by the patient. Therefore, these costs are underestimates of the true costs associated with these events. Extrapolating these cost estimates to the national level and assuming that 78–304 persons were misdiagnosed and treated in 2000, we estimate that the preventable costs to the U.S. health-care system were $3.35 million to $13.06 million in 2000.

To examine the potential to predict laboratory cross-contamination or mislabeling, Jasmer et al. (16) established reproducible and predetermined criteria on the basis of DNA fingerprinting. They prospectively reviewed these events in three large, experienced laboratories in California. Laboratory procedures were reviewed at the start of the study; culture-positive results for 6 (2%) of 296 persons were caused by laboratory cross-contamination, which could be identified a priori. In this study, five of the six persons received unnecessary, expensive, and potentially dangerous medical treatment.

## Monitoring Interjurisdictional Transmission

The impact of epidemic spread of TB across state or jurisdictional boundaries and the necessity for interjurisdictional public health collaboration are not always fully accepted or appreciated, due in large part to the constitutionally mandated independence of state governments for public health practice. Many reports of localized-only epidemic transmission (17) suggest that most transmission of TB in the United States occurs locally. Indeed, Ellis et al. (18) show that most clusters (66% or 680/1,029) from the National Tuberculosis Genotyping Surveillance Network were restricted to a single site.

However, a few recent reports illustrate the importance of the wider geographic spread of TB and the necessity for interjurisdictional collaboration (19,20). Ellis et al. also show that 260 (25%) clusters from the genotyping surveillance network were found in two sites, 55 (5%) in three, 19 (2%) in four, 8 (1%) in five, and 7 (1%) in six sites. As expected, clusters that spanned multiple sites included more case-patients. Maximum cluster size and absolute numbers of case-patients with isolates that clustered continued to increase through the end of the study. Though many of the case-patients in clusters that spanned these multiple sites may not be epidemiologically linked, other examples of interjurisdictional transmission exist (19–22).

We know that TB is highly problematic in certain groups at high risk (i.e., homeless persons) (23–29). With this in mind, an exception to the concept of local-only spread is described by Lathan et al. (30). They present data showing that interjurisdictional collaboration in TB control was necessary to control epidemic spread between adjacent jurisdictions (i.e., Maryland with Washington D.C.). Through combined DNA fingerprinting cluster analyses, these researchers found additional and unsuspected TB transmission that not only crossed state lines but also crossed social lines (i.e., between homeless and nonhomeless persons).

McElroy et al. (31) show the value of a system to compare DNA fingerprinting data across jurisdictions, especially during multistate TB outbreak settings. Facilitating an interjuridictional investigation of TB first recognized in Maryland, they extended the investigation outside of Maryland and discovered an additional 18 case-patients linked to the original 21 previously described.

TB control programs that employ DNA fingerprinting should work with neighboring jurisdictions to devise strategies that promote rapid sharing of results. Comprehensive interjurisdictional monitoring of transmission would require a national registry of DNA fingerprinting data and a system to alert public health officials about interjurisdictional clustering.

## Program Evaluation

Understanding the transmission characteristics of multidrug-resistant TB strains is essential for developing successful control strategies. Multidrug-resistant TB is an important problem and represents a life-threatening condition; patients often require prolonged treatment and frequent hospitalization. The potentially serious side effects of second-line TB drugs challenge effective treatment and the resources of the patient and the health-care system (32).

One indicator for the success of a TB control program’s performance is a decline in TB incidence rate over time. Incident TB case-patients include those recently infected who progress rapidly to active TB and those with remote infection who progress after years of latency and are later diagnosed with active TB. However, in high transmission circumstances, recent disease transmission likely accounts for most incident TB case-patients. DNA fingerprinting clustering may reflect recent TB transmission (4,5,33,34), albeit with methodological and population characteristic caveats (35–37). Therefore, the decline in the incidence of clustering can indicate the impact of interventions aimed at reducing recent TB transmission (38).

Munsiff et al. (39) describe how DNA fingerprinting, specifically molecular clustering of multidrug-resistant TB strains as a surrogate of recent transmission, was used to monitor an improved TB program in New York City*.* During a 3-year period (1995–1997), multidrug-resistant TB was diagnosed for 241 case-patients (4.9% of all culture-positive, TB case-patients) in New York City. These 241 case-patients were more likely than culture-positive, non– multidrug-resistant TB case-patients to have been born in the United States, to be infected with HIV, to be health-care workers, and to have positive acid-fast bacilli smear results**.** During this study period, of 234 multidrug-resistant TB case-patients with DNA fingerprinting results, 153 (65%) were grouped into 19 clusters. Epidemiologic links were identified for 25 (19.8%) case-patients clustered by DNA fingerprinting.

Kong et al. (40) demonstrate the role of DNA fingerprinting to measure the performance of a tuberculin skin-test program among homeless persons. They showed a decrease in clustering (a surrogate of recent TB transmission) from 49% during the 7-year period before the program was implemented to 14% in the 4-year period after the program. This assessment is a logical extension of the usefulness of DNA fingerprinting technology, since a previous report by Burman et al. (41) showed that homelessness was a predictor of DNA fingerprint clustering.

Ellis et al. (18) show that, despite a decrease in TB incidence rates at all genotyping surveillance network sites, the proportion of cases in clusters stabilized at a relatively high level (~48%). They suggest that this high proportion of clustering may be due to the inclusion of many low-incidence, stable populations in which persons in chains of transmission from past decades still reside in proximity (42). Alternative explanations include a slightly younger population under study, the short length of the observation period, the presence of old endemic strains that have spread widely, and the limited discrimination of low-copy number IS*6110* restriction fragment length polymorphism (RFLP) patterns, even with the addition of spoligotyping as a secondary test. Although the overall proportion of case-patients in clusters plateaued at approximately 48% over the 5 years of study, the annualized proportion of case-patients in clusters decreased over time with a concomitant decrease in TB incidence. This finding, which might reflect more effective TB control in limiting ongoing transmission, is provocative and merits further investigation.

## Statewide Assessment of Circumstances and Settings for TB Transmission

The statewide use of DNA fingerprinting provides an informed picture of the epidemiologic features of disease transmission (36–38). Cronin et al. (43) describe a specific example in Maryland. During a 5-year period, they used DNA fingerprinting on >99% of all isolates in the state and found that cluster investigations were very effective in identifying additional epidemiologic links, many of which occurred in nontraditional settings. Specifically, isolates from 436 (37%) of 1,172 Maryland case-patients were clustered by DNA fingerprinting. Of those 436 clustered case-patients, these researchers found 155 (36%) to be epidemiologically linked using traditional contact investigation.

Miller et al. (44) provide another example of statewide use of DNA fingerprinting by exploring the impact of DNA fingerprinting used in Massachusetts during investigations of a TB outbreak and a laboratory cross-contamination event. These researchers also describe how DNA fingerprinting affected the identification of *M. tuberculosis* strains and transmission sites and accurate epidemiologic links. Overall, they found that, in addition to 129 epidemiologic relationships found before DNA fingerprinting results were obtained, 12 other epidemiologic relationships involving 20 persons were discovered as a result of cluster investigations. In addition, they determined places of transmission previously unrecognized and used DNA fingerprinting to refute a purported TB outbreak.

Sharnprapai et al. (45) also report on the use of DNA fingerprinting to further understand the epidemiology and transmission patterns of TB in Massachusetts. In this study, 28% of TB case-patients were clustered, and case-patients born in the United States were two times more likely to cluster than case-patients not born in the United States. Furthermore, they point out a very important limitation to interpreting and using DNA fingerprinting data. Despite using a secondary typing method (spoligotyping) with strains that have six or fewer copies of IS*6110*, a limited ability to differentiate these strains exists. In their study, clusters of strains with more than six copies of IS*6110* were more likely to have epidemiologic links found than clusters of strains with six or fewer copies of IS*6110*.

The report by Dillaha et al. (46) describes the subtleties of disease transmission faced by TB programs in low-incidence states (specifically Arkansas), which are in the forefront as the United States moves toward TB elimination. Thirty-five case-patients in a 54-year period with identical or very similar fingerprints were identified. After reviewing the endemic strain, these researchers recognized the lack of success with traditional contact tracing and treatment recommendations for latent TB infection for persons with positive tuberculin skin tests. This critical determination has implications for other low-incidence areas. In addition to the traditional focus on personal contacts, Dillaha et al. recommend case finding and screening on the basis of geographic location. With the advent of geographic interface technology and mapping, this new public health strategy might be feasible. Additionally, these findings support the usefulness of a social network approach to contact investigation.

## Outbreak Investigation

The value of DNA fingerprinting has been shown clearly during outbreak investigations (1–3). During every investigation, one overriding question recurs: which case-patients related to the outbreak are part of the chain of transmission and are not unrelated, sporadic cases? DNA fingerprinting can help researchers determine whether patients are related to the outbreak and thus focus the epidemiologic investigation.

An intriguing new benefit of coupling DNA fingerprint information with outbreak investigation lies in the power of this tool to increase understanding of the often difficult to discern transmission patterns of community TB disease (47) and uncover previously unknown outbreaks. Ijaz et al. (48) show this potential use by demonstrating that molecular clusters could show previously unsuspected instances of probable TB transmission, prompting more directed investigations to seek epidemiologic links missed by routine contact investigation. In the study, cluster analysis was based on identical and similar DNA fingerprinting patterns, broadening the group of patients included in the initial analysis.

In their study, secondary typing was accomplished by using a polymorphic GC-rich sequence for identical IS*6110*-based DNA fingerprinting patterns with fewer than six bands or for patterns with more than six bands that were similar but differed by a single band (49). In further investigations of clusters with this “broader net,” among 66% of case-patients, Ijaz et al. uncovered additional epidemiologic links missed during routine contact investigations in Arkansas. During this process, they found an extensive, previously unknown social network that aided public health investigations. Ijaz et al. conclude that patients whose isolates have similar but not identical IS*6110* patterns should be considered potential members of a cluster and be included during epidemiologic investigations.

Oh et al. (50) help establish the value of DNA fingerprinting in an unusual outbreak setting, a zoo. They describe a multispecies epizootic with genotypically identical *M. tuberculosis* strains*.* Their DNA fingerprint investigation showed that five of six animals had the identical strain and that zoo employees with previous negative tuberculin skin tests were exposed. Skin tests for 55 (18%) of 307 employees were positive, showing evidence of recent infection.

Bennett et al. (51) present data from the genotyping surveillance network with important policy implications. In addition to indicating that contact investigation should be extended to all settings frequented by the source case-patient, they also showed a significant positive association between being a smear-negative source case-patient and having unconfirmed transmission. This finding suggests that the identification of a smear-negative source case-patient (as an index case-patient) should not preclude the ongoing investigation for other possible sources. They also suggest that transmission from smear-negative case-patients is not negligible.

Sun et al. (52) report data gathered on transmission of tuberculosis to children <5 years of age. Representing a sentinel health event, thorough investigation of the circumstances of childhood tuberculosis remains critical to effective public health practice. They found that routine public health investigations conducted by local health departments, within the National Tuberculosis Genotyping Surveillance Network, identified suspected source patients for 57 (51%) of 111 culture-confirmed case-patients <5 years of age. For 8 (15%) of these 57 patients, DNA fingerprinting suggested infection with different strains. These children were more likely to be older than other children and source case-patients with identical strains. The findings in this study highlight the requirement of rigorous case and contact investigation efforts, especially in household settings.

## DNA Fingerprinting Laboratory Techniques

Other articles in this issue present data that improve our understanding of both laboratory facets of DNA fingerprinting (i.e., IS*6110* fingerprinting) and basic science of *M. tuberculosis.* Braden et al. (53) report the results of an external quality assessment program for the seven network laboratories in which the interlaboratory reproducibility was measured. They found that, overall, an exact match was achieved for 73% of isolates in panels: 90% matched with a one-band difference and 97% matched with a two-band difference. Although they report that final outcomes of pattern analysis and cluster determination in the genotyping surveillance network were probably closer to reality than the results of this quality assurance exercise suggest, they also warn that the variability and nonreproducibility are substantial and should be considered when interpreting the results from the genotyping surveillance network. Crawford et al. (54) demonstrate through the establishment of the genotyping surveillance laboratory, that DNA fingerprinting remains an “art,” and the experience and training of laboratorians are important.

Driscoll et al. (55) describe an evaluation of “logo analyses.” Array-based assays use reverse hybridization. The binary nature of array-based assays allows data to be analyzed usefully with algorithms associated with motif recognition, such as sequence logo analyses. Logo analyses have the potential to aid in visualizing and displaying spoligotyping cluster data and in managing the enormous amount of digital data generated by large-scale DNA fingerprinting projects. This potential is especially relevant now because low-incidence states and countries (including the United States) are considering universal implementation of DNA fingerprinting of *M. tuberculosis*. Using these and other bioinformatic tools, scientists will be able to interpret and understand the data generated by such a project.

Lok et al. (56) demonstrate that secondary typing methods (e.g., spoligotyping) should be used when isolates have no IS*6100* insertions (i.e., zero-band strains). This article describes the differentiation power of secondary typing in these instances. In a second paper, Lok et al. (57) demonstrate the use and power of polymerase chain reaction techniques (e.g., variable number of tandem repeats) to distinguish and characterize the most common *M. tuberculosis* strain pattern in the United States—a two-band IS*6110* RFLP pattern representing 5% of all isolates in the National Tuberculosis Genotyping Surveillance Network.

## Diversity of *M. tuberculosis* Strains

The second important implication for DNA fingerprinting of *M. tuberculosis* is its ability to measure the overall diversity of *M. tuberculosis* strain patterns, including differences within the United States, differences by region and population, and prevalence of endemic strains. The genotyping surveillance network database demonstrated this diversity in the United States (57). The 10,883 patients in the study represent approximately 11.6% of all new TB cases in the United States from 1996 through 2000. Through this study, DNA fingerprinting of 10,883 isolates was performed by using the IS*6110* RFLP method, yielding 6,128 distinct patterns.

Cowan et al. (58) report that family analysis of IS*6110* patterns revealed 497 patterns related to the W-Beijing family (19); these patterns represent 946 isolates or 9% of all isolates in the genotyping surveillance network. Six new families of related DNA fingerprint patterns were also proposed for isolates containing 6–15 copies of IS*6110*. These families contain up to 251 patterns and 414 isolates; together, they contain 21% of isolates in this copy-number range and may represent endemic strains distributed across the United States.

The 8,245 isolates with more than six copies of IS*6110* yielded 5,640 fingerprint patterns. Of these, 4,846 (86%) were identified for a single isolate, and 794 patterns grouped 3,399 isolates into fingerprint-defined clusters. Of 457 fingerprint patterns identified among the 2,507 isolates with low-copy numbers (six or fewer copies of IS*6110*), 314 (69%) were reported for a single isolate, and 143 grouped 2,193 isolates into clusters. Clustering was much greater among isolates with low-copy numbers (87%) than among isolates with high-copy numbers (41%).

## <H2>Limitations, Challenges, and Future Considerations

The “state of the art” in applied DNA fingerprinting technology has scientific and molecular limitations, as well as stumbling blocks to practical use in the field. The lack of reproducibility of the RFLP DNA fingerprinting technique and the difficulty in comparing patterns in an RFLP DNA fingerprint database remain important limitations in developing strategies for universal implementation. The other important factor related to RFLP is the time required for obtaining results. In order for DNA fingerprinting to provide value to routine contact investigations, these molecular data must be available on a timely basis, so that public health intervention specialists can use them in cluster investigations.

Furthermore, the precision of the interpretation these data is evolving. Clustering, by itself and in its entirety, is not always equivalent to recent transmission; however, a portion of it is. The limitation of clustering interpretation must be scientifically established, especially if that interpretation is used as a marker for public health practice performance and as an indication of progress toward elimination of TB. We know that the population under study and the length of observation time play important roles in interpreting this measure. In addition, the specificity of the clustering case-definition factors into this equation. Additional investigation into this area is necessary.

How well these research techniques can be expanded to assist TB control programs is not clear. Laboratory programs must be established to provide understandable, real-time results in a manner that influences decisions. The expansion of these techniques to assist TB control programs holds great promise. However, cluster investigation must be incorporated into routine public health practice, including the standardization of protocols. TB control officials require further training to interpret DNA fingerprinting results and act on these results in an effective way. The National Tuberculosis Controllers Association, in collaboration with the CDC, is currently drafting a DNA fingerprinting handbook to help health workers in the field understand and interpret DNA fingerprinting data.

We intend to continue analyses of the National Tuberculosis Genotyping Surveillance Network data to gain additional insight into the value of cluster investigations. If DNA fingerprinting is to be implemented universally, this approach should be flexible enough to adapt to future laboratory techniques, as they become available. We think that DNA fingerprinting will become an essential tool in investigating TB transmission in difficult populations and unusual circumstances; consequently, DNA fingerprinting will be vital in the effort to eliminate TB.
